# Facts Worth Remembering

**Published:** 1882-07

**Authors:** 


### FACTS WORTH REMEMBERING.

Sudden Deaths do not come from heart disease, one case in twenty, but from congestion of the lungs or brain, or from apoplexy. More die from congestion of the lungs than of the brain, and more of congestion of the brain than from apoplexy.

Sudden death from heart disease is usually caused by rupture of some large artery near the heart ; from congestion of the lungs, by instantly stopping the breath ; from congestion of the brain, by causing pressure on the brain, which paralyzes and instantly destroys life ; from apoplexy, by hemorrhage in the brain.

Heart disease most frequently results from neglected or improperly-treated rheumatism. It more often follows mild rheumatism than the severe kind, because severe rheumatism receives prompt treatment, while the mild form is often neglected and left to work its way to the heart.

Persons who suppose themselves suffering from heart disease, because they have pain in the region of the heart, or palpitation, seldom have any disease of that organ. In nine cases out of ten, they are sufferers from dyspepsia—nothing more. Congestion of the lungs is most frequently caused by a sudden change from the heat of an ill-ventilated room or railroad car, or street car, to the cold air outside, without being protected by sufficient clothing ; hence, many persons thus seized drop dead in the streets.

Congestion of the brain most frequently results from trouble and anxiety of mind, producing sleeplessness, followed by engorgement of the small blood vessels of the brain, sudden loss of vital power, and almost instant death. Apoplexy may be an inherited disease, or it may be induced by too free living, or its opposite, too great abstemiousness. Paralysis may affect only a small portion of the body, from a finger or toe to an entire limb, or it may disable half the body, or the whole body, when death soon follows. When half the body is affected by paralysis, we may be certain that the seat of the disease is in the opposite side of the brain, because nerve fibres cross. Partial paralysis is often temporary when caused by the rupture of a small blood vessel, if the clot is got rid of by absorption or otherwise.

Although this is a disease that all classes of people are liable to, its most destructive work is done among the depraved and dissipated. There is no doubt that the habitual use of tobacco is one of the most prominent causes of paralysis and other nerve diseases.

A Severe Cold can be soonest cured by remaining within doors, in a warm room and near the fire, until all signs of it have disappeared. Then, care should be taken to prevent a relapse by having the feet warmly clad, and the whole body, and particularly the chest and the back of the neck, well protected when going out.

A Recent Cough will almost always yield to the following treatment within two or three days : Mix in a bottle 4 ounces of glycerine, 2 ounces of alcohol, 2 ounces of water, 2 grains of morphine. Shake well. Dose for an adult, 1 to 2 teaspoonfuls every 2 or 3 hours. Half this quantity to children from 10 to 15 years. It is not safe to give it to infants or children under 10 years of age.

To Stop Bleeding, if from a cavity in the jaw after a tooth has been extracted, shape a cork into the proper form and size to cover the bleeding cavity, and long enough to be kept firmly in place when the mouth is closed. This, we believe, is our own invention, and we have never known it to fail. It has served us in desperate cases.

When an Artery is Cut, the red blood spurts out at each pulsation. Press the thumb firmly over the artery, near the wound, and on the side toward the heart. Press hard enough to stop the bleeding, and wait till a physician comes. The wounded person is often able to do this himself, if he has the requisite knowledge. The main object of this "Journal" is to teach its readers, not only how to protect themselves from diseases, but how to save life in accidents and emergencies.

Simple Fractures may be adjusted by almost any one. Get the limb as near as possible in the natural position, and then send for a doctor. There is no great urgency in such cases.

In Fracture of the Skull, with compression and loss of consciousness, examine the wound, and, if possible, raise the broken edges of the skull so as to relieve the pressure on the brain. Prompt action would often save life.

In Case of Poisoning, the simple rule is to get the poison out of the stomach as soon as possible. Mustard and salt act promptly as emetics, and they are always at hand. Stir a tablespoonful in a glass of water, and let the person swallow it quickly. If it does not cause vomiting in five minutes, repeat the dose. After vomiting, give the whites of two or three eggs, and send for the doctor.

Burns and Scalds are soonest relieved by an application of cold water. Dry carbonate of soda, or baking soda, sprinkled over the burned spot, is the latest remedy, and is said to be very effectual.

These means are only temporary. In severe cases, a physician should be sent for.

